# Determinants of Postnatal Care Check-ups in Ethiopia: A Multi-Level Analysis

**DOI:** 10.4314/ejhs.v31i4.9

**Published:** 2021-07

**Authors:** Afework Tadele, Masrie Getinet

**Affiliations:** 1 Population and Family Health, Jimma University, Jimma, Ethiopia; 2 Epidemiology and Biostatistics, Jimma University, Jimma, Ethiopia

**Keywords:** Postnatal care, multilevel, EDHS, Ethiopia

## Abstract

**Background:**

Postnatal care is provided to women and their babies within 42 days after delivery. Although the first two days after birth was a critical time in maternal health, it was the most neglected period of maternal health services. Therefore, this study aims to determine the maternal and community-level factors of postnatal check-ups in Ethiopia

**Methods:**

Ethiopian Demographic and Health Survey (EDHS) in 2016 was utilized. A total of 3,948 women aged 15–49 giving birth in the two years before the survey were included. A multi-level mixed-effects logistic regression model was employed.

**Result:**

Only 17% [95% C.I; 16.46%–17.53%] of the women had a postnatal check-up (PNC) within 2 days of giving birth in Ethiopia. Institutional delivery AOR 2.14 [95% C.I 1.70, 2.0] and giving birth by cesarean section AOR 1.66 [95% CI 1.10, 2.50] were found to be maternal factors. Whereas, administrative regions (Oromia 69%, Somali 56%, Benishangul 55%, SNNPR 43%, Gambela 66%, Afar 50% and Dire Dawa 55% which less likely to utilize PNC as compared to Addis Ababa), higher community-level wealth AOR 1.44 [95% C.I 1.08, 1.2], ANC coverage AOR 1.52 [95% C.I 1.19, 1.96] and perceived distance of the health facility as a big problem AOR 0.78 [95% C.I 0.60, 0.99] were the community level factors.

**Conclusion:**

Both maternal factors and community factors are found to be a significant association with PNC, however, based on the ICC maternal factors prevail the community-level factors. Therefore, public health interventions to increasing improve postnatal care services should focus on community level determinants.

## Introduction

The postnatal period begins immediately after childbirth and lasts six weeks (1). It is an essential element across the continuum of care in the maternal and child health care services, and serve as a gateway for family planning services. As a result, woman and her partner/family require more information than they usually receive on the care of the baby and mother within the first week after childbirth, to stay safe from maternal and neonatal complications (2). Nowadays, about 810 women die globally from preventable causes of pregnancy and child birth a day. Low and Lower-Middle-Income (LMIC) countries account for 94% of the death (3). Moreover, evidence revealed that most of the maternal death recorded in the early postnatal period. For instance, more than 80% of the mortality in the first fourteen days after birth, and more than 60% in the post-natal period in both developing countries and the United States (4). Yet, this is the most neglected period in the provision of quality care (5).

Although there is a good improvement in maternal and child health care services coverage recently, less than one in five women received a postnatal check within the first two days of birth in Ethiopia (6). Thus, the experiences and expectations of women and their families, and barriers to the uptake of services and/or access to services also should be considered for improving postnatal care services in the country (1).

Previous work in Ethiopia has only focused on identifying the association of maternal characteristics like education, wealth (7,8), residence, occupation, Antenatal Care (ANC) follow-up, institutional delivery, (9–15), and distances from health institutions (16) with postnatal check-ups. Although this approach is interesting, it fails to consider the community-level variables. Postnatal care clients were living in a community with different social contexts. Therefore, this study provides a great input to design different programs, policies and strategies on postnatal check-ups. Therefore, the aim of this study is to determine maternal and community level determinants of postnatal check-ups in Ethiopia.

## Methods

**Data source:** The dataset utilized in this study was obtained from the DHS program accessed from http://dhsprogram.com/data/.

In this study, 3984 women aged 15–49 years who gave birth in the two years before the survey, were included.

**Study variables definitions and measurements**

**Dependent variable**: If a woman received postnatal check-ups within two days of delivery, she was categorized as received a postnatal care service (coded as 1) and otherwise not received postnatal check-ups (coded as 0).

**Independent variables**: The independent variables for postnatal care use were broadly classified into maternal characteristics and community-level variables in line with a multilevel analytic approach.

**Maternal variables**: educational level of women, household wealth index, perceived distance to a health facility to get medical help, employment status of women's, number of ANC visits during pregnancy, delivery by caesarean section, birth order, and place of delivery were included as maternal-level variables.

**Community-level variables**: Community-level wealth index, community-level ANC coverage, community-level women's education, perceived distance to a health facility to get medical help at the community level were considered as community-level variables.

**Region**: The EDHS sample was collected from nine regions and two administrative cities. We used these administrative boundaries as community-level factors.

**Place of residence**: It was classified as rural (coded as 1) and urban (coded as 0), considered as a community level factor.

**Community-level education of women**: The median value of educational attainment at the national level was 5 years. Thus, the median value of the aggregated clusters below 5 was classified as low education (coded as 0) of women and the median value of the aggregated clusters 5 and more were classified as high education (coded as 1) of women.

**Community-level wealth**: Similarly, the median value of the wealth index at the national level was 3. Then, the aggregated clusters were classified as low wealth (coded as 0) and high wealth (coded as 1) by considering the national value as a cut-off point.

**Perceived distance to a health facility at the community level**: At the national level the proportion of perception of distance to a health facility to get medical help as a big problem was 0.45. So, clusters were classified as low perception (coded as 0) and high perception (coded as 1) using the national proportion as a cut-off point after aggregated of it.

**Community-level ANC visit:** The median value of ANC visit at the national level was three. Hence, the median value of the cluster less than three ANC visits was classified as low (coded as 0) and greater than or equal to three ANC visits were classified as high (coded as 1) after aggregated the ANC visit.

**Data analysis**: Two-level mixed-effects logistic regression analyses were employed using STATA version 14. Since 2016 EDHS data was hierarchical, i.e., individuals (women) were nested in households, and households were nested in the cluster. The unit of analysis for the characteristics of community-level factors was the cluster. For this study, we included 645 clusters in which all the women who most recent birth was within two years preceding the survey resides.

First, bi-variable two-level mixed-effects logistic regression analyses were done to assess the association between the independent variables and the dependent variable of the study. The overall categorical variables with a p-value of <0.25 at the bivariate two-level mixed-effect logistic regression analysis were included in the final model of the multivariable two-level mixed-effect logistic regression model in which odds ratio with 95% confidence intervals were estimated to identify the independent variables of postnatal check-ups. P-values less than 0.05 were employed to declare statistical significance. Fixed effect and random effect were calculated to assess the maternal and cluster variations respectively. Moreover, the frequency table was displayed for the maternal and community level variables. All analysis was done on weighted data.

In this analysis four models are displayed, null model (model containing no factors), a model I (containing only maternal factors), model II (containing only community factors), and model III (both maternal and community-level factors).

The intra-class correlation (ICC) was calculated as the proportion of the between cluster variation in the total variation.

The variability on the odds of postnatal check-up explained by successive models was calculated by Proportional Change in Variance (PCV).

A two-level mixed-effects logistic regression model was used to analyze the effects of community characteristics and women's individual-level factors in postnatal care services utilization in Ethiopia.

As depicted in the empty model, 36.5% of the variations in postnatal check-ups could be attributed to community characteristics. The value of the log-likelihood results consistently decreased as fitted models progressed from the empty model to Model 1, Model 2, and Model 3 indicating that the fitted models were a better fit to the data (Table 1). The higher the ICC, the more relevant were the community characteristics for understanding individual variation in postnatal check-ups for mothers. Accordingly, the combined model of maternal, and community factors were selected for determining postnatal check-ups in Ethiopia.

**Table 1:** Multilevel logistic regression model showing random-effects on postnatal check-ups in Ethiopia: Random effects

	Model 0	Model 1	Model 2	Model 3
ICC (%)	36.5%	7.1%	6.8%	1%
PCV	Reference	81%	81.4%	97%
Model fitness				
Log likelihood	-1883.6	-1736.0	-1633.2	-1536.0

## Results

**Maternal characteristics**: The majority (61.09%) of the women who gave birth in the two years preceding the survey, did not attend formal education. More than seventy-five percent of the women were unemployed. Still, there is a great problem of access to a health facility in Ethiopia as the majority (61.43%) of the women's perceived distance to a health facility to get medical help was a big problem. Regarding maternal health services in the last two years, only 32.62% percent of the women attended four and more ante-natal care and 35.22% percent gave birth in a health facility (Table 2).

**Table 2:** Socio-demographic and obstetric characteristics of the women aged 15–49 giving birth in the 2 years in Ethiopia (n=3948)

Maternal variables	Categories	Frequency	% Weighted
**Residence**	Urban	767	11.34
	Rural	3181	88.66
Educational level of women	No education	2373	61.09
	Primary	1086	30.34
	Secondary	323	5.83
	Higher	166	2.74
Employment status	No	2958	75.23
	Yes	990	24.77
Number of ANC visit	<4	2527	67.38
	4^+^	1421	32.62
Delivery by cesarean section			
	No	3818	97.4
	Yes	130	2.60
Place of delivery	Home	2382	64.78
	Health facility	1566	35.22

**Community-level characteristics**: More than eighty-eight percent of the clusters were from rural areas of Ethiopia, while sixty percent of them were classified under higher community-level wealth status. There is also about 75% community level of antenatal care coverage and women's unemployment status revealed. One in three clusters perceived the distance to Health facility to get medical help at the community level is a big problem (Table 3).

**Table 3:** Community level characteristics of the women aged 15–49 giving birth in the 2 years in Ethiopia (n=3948)

Maternal variables	Categories	Frequency	% Weighted
**Community-level wealth**	low	2017	40.33
	High	1931	59.67
**Community-level ANC coverage**			
	low	2687	74.95
	High	1261	25.05
**Community-level women's employment**			
	low	2854	75.49
	High	1094	24.51
**Community-level women's education**			
	low	1874	61.43
	high	1541	38.57
**Perceived distance to** **Health facility** **to get medical help at the** **community level**	Not a big problem	1506	30.02
	Big problem	2442	69.98

**Independent predictors of postnatal check-ups in Ethiopia**: The details of the effect sizes of both individual and community-level factors on the odds of postnatal care service utilization are described in Tables 4. Delivery by caesarean section was independently and significantly associated with postnatal care utilization. After adjusting for maternal and community-level factors, the odds of using postnatal care was 1.66 times OR 1.66 (95% CI 1.10, 2.50) higher among women gave birth by caesarean delivery compared to their counterparts. Similarly, women who gave birth at health facilities were twice higher odds of using postnatal care OR 2.14 (95% CI 1.70, 2.70) as compared to those delivered at home (Table 4)

**Table 4:** Multilevel mixed-effect logistic regression results of maternal and community-level factors associated with timely use of postnatal care in Ethiopia, 2016 EDHS

Variables/ characteristics		Null model	Model I	Model II	Model III

			Maternal characteristics	Community-level characteristics	Maternal and community-level characteristics
			OR (95%CI)	OR (95%CI)	OR (95%CI)
Delivery by cesarean section					
	**No^(ref)^**		1		
	**Yes**		1.76(1.18, 2.63) **		1.66(1.10, 2.50) *
Place of delivery					
	**Not at a health facility**		1		
	**Health facility**		2.84(2.27, 3.55) ***		2.14(1.70, 2.70) ***
Administrative regions					
	**Tigray**			1.31(0.82, 2.10)	1.35(0.88, 2.07)
	**Afar**			0.38(0.21, 0.68) **	0.50(0.29, 0.87) *
	**Amhara**			0.49(0.29, 0.83) **	0.63(0.39, 1.03)
	**Oromia**			0.24(0.14, 0.42) ***	0.31(0.19, 0.50) ***
	**Somali**			0.32(0.18, 0.56) ***	0.44(0.26, 0.73) **
	**Benishangul**			0.36(0.21 0.63) ***	0.45(0.28, 0.79) **
	**SNNPR**			0.47(0.29, 0.78) **	0.57(0.36, 0.90) **
	**Gambela**			0.27(0.15, 0.48) ***	0.34(0.20, 0.58) *
	**Harari**			1.06(0.62, 1.80)	1.10(0.68, 1.79)
	**Dire Dawa**			0.49(0.29, 0.83) **	0.55(0.34, 0.89) *
	**Addis Ababa^(ref)^**			1	
Community-level wealth					
	**low^(ref)^**			1	
	**High**			1.60(1.21, 2.13) **	1.44(1.08, 1.2) *
Community-level ANC coverage					
	**low^(ref)^**			1	
	**High**			1.73(1.34, 2.25) ***	1.52(1.19, 1.96) **
Community-level perceptions of distance to HF					
	**Not a big** **problem^(ref)^**			1	
	**Big problem**			0.70((0.54, 0.90) **	0.78(0.60, 0.99) **

* P<0.05

** P<0.001

*** P<0.0001

There is significant administrative regional variation in postnatal care use. The conspicuous observation to emerge from the data comparison was in Oromia 69%, Somali 56%, Benishangul 55%, SNNPR 43%, Gambela 66%, Afar 50% and Dire Dawa 55% which less likely to utilize postnatal care utilization as compared to Addis Ababa city administrative area. While there is no significant difference between residents of Harari city, Amhara regional state, and Tigray regional state as compared to Addis Ababa city.

Community-level wealth and antenatal care coverage were also found to be significant determinants of postnatal care utilization. Community-level wealth was l.4 times OR 1.44 (95% C.I 1.08, 1.2) and community level antenatal care coverage was 1.5 times OR 1.52 (95% C.I 1.19, 1.96) more likely to use postnatal care utilization.

Perceived community level distance of health facility as a big problem was found to be highly significant determinants of postnatal care. A community who perceived the distance to a health facility as a big problem were 22% less likely OR 0.78 (95% C.I 0.60, 0.99) to utilize postnatal care as compared to their counterparts (Table 4).

## Discussion

Identifying maternal characters and community-level determinants of postnatal care utilization has a great contribution in designing different interventions for improving maternal and child health. Therefore, this study employed multilevel mixed effect modelling to identify maternal and community-level determinants of postnatal care check-ups in Ethiopia.

This study found that women who gave birth by caesarean delivery were 1.66 times higher odds of using postnatal care services within the first two days compared to those given birth by another mode of delivery. This is in good agreement with the study in rural Tanzania in 2015 revealed cesarean section delivery was positively associated with postnatal care use (17). This might be women who gave birth by cesarean section stay at the facility for about two days for which they receive a postnatal check-up.

In another way, women who gave birth at the health facilities were twice more likely to utilize postnatal care as compared to those delivered at home. This substantiates previous findings in the study in different parts of Ethiopia like Tigray and SNNPR, (9–11, 17) and Tanzania (17). This might be clients who visit the health facility would have different health-seeking behaviors with those never attended health facility.

There is significant administrative regional variation in postnatal care use in Oromia, Somali, Benishangul, SNNPR, Gambela, Afar, and Dire Dawa which less likely to utilize postnatal care utilization as compared to Addis Ababa city administrative area. While there is no significant difference between residents of Harari city, Amhara regional state, and Tigray regional state as compared to Addis Ababa city. Variation in postnatal care service utilization varies was also observed in different parts of Ethiopia (8–10, 18, 19) and West African countries (20). There are several possible explanations for this finding, first the difference in geographic accessibility of the postnatal care services due to topography and unfavorable roads for the mothers in rural areas of Ethiopia; second, there was a difference in local cultures and beliefs in different areas of the country; third, the difference in urbanized geographical areas among different regions.

Being in the higher community level antenatal care coverage was 1.5 times more likely to utilize post-natal care services. Our findings appear to be well supported by a multilevel analysis of DHS in sub-Saharan Africa in 2014, which found significant associations between four or more antenatal care visits and ever breastfed with both outcomes (21). The results point to the likelihood of information diffusion for postnatal care utilization in the community.

Being a resident of higher community-level wealth was l.4 times more likely to utilize post-natal care services. This concurs well with the study in West Africa in 2018, which revealed community-level poverty was a significant determinant of postnatal care use (20). This would appear to indicate that a wealthy community was more probability of getting health information and reside in urban areas.

A community who perceived the distance to a health facility as a big problem were 22% less likely to utilize postnatal care as compared to their counterparts. We believe that no other authors have found that postnatal care utilization is less likely in the community who perceived the distance of health facility as a big problem. There is a good probability that community telling can influence any health care service utilization.

This study provides strong evidence in utilizing community-based representative data of DHS and the use of multilevel mixed-effects analysis which bring disaggregated data on individual characteristics and community-level determinants for designing contextual interventions.

We are aware that our research may have three limitations. The first is findings are based on quantitative data only that cannot explore the detailed reasons in the community for low levels of postnatal care use. The second is excluding men's and other community-level significant others view, may not give fully address the community-level determinants. Lastly, the information on postnatal care provided by mothers was retrospective and those women who had given birth 2 years ago can't probably remember accurately the service they received. These limitations are evidence of the DHS women's data not inclusive of the above problems.

In conclusion, less than one in five women utilized postnatal care in the first 2 days after birth in Ethiopia. This is unacceptably very low. Giving birth institutionally and by cesarean section are found to be maternal factors, whereas administrative regions, community-level wealth, community-level antenatal care coverage and perceived distance of the health facility as a big problem were community-level determinants. This indicates both maternal factors and community factors are found to be a significant determinant of postnatal check-ups. However, based on the ICC maternal factors prevail the community-level factors.

Therefore, public health interventions to increasing improve postnatal care services should focus on community level determinants specifically women reside in the community with lowest wealth status, antenatal care coverage and living remote areas from health facility. Federal Ministry of Health's maternal health directorate should realize the equity in postnatal care services among administrative regions of the country. Exploring community-level determinants in different areas of the country using qualitative data with the inclusion of male responses were recommended for scientific communities.

## References

[1] World health organization (2010). Technical Consultation on Postpartum and Postnatal Care.

[2] Kikuchi K, Yasuoka J, Nanishi K, Ahmed A, Nohara Y, Nishikitani M (2018). Postnatal care could be the key to improving the continuum of care in maternal and child health in Ratanakiri, Cambodia. PloS one.

[3] WHO (2018). Maternal mortality: fact sheets.

[4] Li XF, Fortney JA, Kotelchuck M, Glover LH (1996). The postpartum period: the key to maternal mortality. International Journal of Gynecology & Obstetrics.

[5] World health organization (2013). WHO recommendations on Postnatal care of the mother and newborn.

[6] Central Statistical Agency (CSA) [Ethiopia] and ICF (2016). Ethiopia Demographic and Health Survey 2016.

[7] Fekadu GA, Ambaw F, Kidanie SA (2019). Facility delivery and postnatal care services use among mothers who attended four or more antenatal care visits in Ethiopia: further analysis of the 2016 demographic and health survey. BMC Pregnancy Childbirth.

[8] Wudineh KG, Nigusie AA, Gesese SS, Tesfu AA, Beyene FY (2018). Postnatal care service utilization and associated factors among women who gave birth in Debretabour town, North West Ethiopia: a community- based cross-sectional study. BMC Pregnancy Childbirth.

[9] Abraha TH, Gebrezgiabher BB, Aregawi BG, Belay DS, Tikue LT, Reda EB (2019). Factors Associated with Compliance with the Recommended Frequency of Postnatal Care Services in Four Rural Districts of Tigray Region, North Ethiopia. Korean J Fam Med.

[10] Abuka Abebo T, Jember Tesfaye D (2018). Postnatal care utilization and associated factors among women of reproductive age Group in Halaba Kulito Town, Southern Ethiopia. Arch Public Health.

[11] Abota TL, Atenafu NT (2018). Postnatal Care Utilization and Associated Factors among Married Women in Benchi-Maji Zone, Southwest Ethiopia: A Community Based Cross-Sectional Study. Ethiop J Health Sci.

[12] Barry D, Frew AH, Mohammed H, Desta BF, Tadesse L, Aklilu Y (2014). The effect of community maternal and newborn health family meetings on type of birth attendant and completeness of maternal and newborn care received during birth and the early postnatal period in rural Ethiopia. J Midwifery Womens Health.

[13] Chaka EE, Abdurahman AA, Nedjat S, Majdzadeh R (2019). Utilization and Determinants of Postnatal Care Services in Ethiopia: A Systematic Review and Meta-Analysis. Ethiop J Health Sci.

[14] Tesfahun F, Worku W, Mazengiya F, Kifle M (2014). Knowledge, perception and utilization of postnatal care of mothers in Gondar Zuria District, Ethiopia: a cross-sectional study. Matern Child Health J.

[15] Tesfaye S, Barry D, Gobezayehu AG, Frew AH, Stover KE, Tessema H (2014). Improving coverage of postnatal care in rural Ethiopia using a community-based, collaborative quality improvement approach. J Midwifery Womens Health.

[16] Darega B, Dida N, Tafese F, Ololo S (2016). Institutional delivery and postnatal care services utilizations in Abuna Gindeberet District, West Shewa, Oromiya Region, Central Ethiopia: A Community-based cross sectional study. BMC Pregnancy Childbirth.

[17] Mohan D, Gupta S, LeFevre A, Bazant E, Killewo J, Baqui AH (2015). Determinants of postnatal care use at health facilities in rural Tanzania: multilevel analysis of a household survey. BMC Pregnancy Childbirth.

[18] Akibu M, Tsegaye W, Megersa T, Nurgi S (2018). Prevalence and Determinants of Complete Postnatal Care Service Utilization in Northern Shoa, Ethiopia. J Pregnancy.

[19] Tesfaye G, Chojenta C, Smith R, Loxton D (2019). Magnitude and correlates of postnatal care utilization among reproductive aged women in a rural district in eastern Ethiopia: A cross-sectional study. Midwifery.

[20] Solanke BL, Amoo EO, Idowu AE (2018). Improving postnatal checkups for mothers in West Africa: A multilevel analysis. Women Health.

[21] Singh K, Brodish P, Haney E (2014). Postnatal care by provider type and neonatal death in sub-Saharan Africa: a multilevel analysis. BMC Public Health.

